# Tetra­kis(2,4,6-triamino-1,3,5-triazin-1-ium) tris­(pyridine-2,6-dicarboxyl­ato)calcate(II) hexa­hydrate

**DOI:** 10.1107/S1600536808022873

**Published:** 2008-07-26

**Authors:** Hossein Aghabozorg, Shirin Daneshvar, Andya Nemati

**Affiliations:** a Faculty of Chemistry, Tarbiat Moallem University, Tehran, Iran; b Department of Chemistry, Islamic Azad University, Ardabil Branch, Ardabil, Iran

## Abstract

The title compound, (C_3_H_7_N_6_)_4_[Ca(C_7_H_3_NO_4_)_3_]·6H_2_O or (tataH)_4_[Ca(pydc)_3_]·6H_2_O (where tata is 2,4,6-triamino-1,3,5-triazine and pydcH_2_ is pyridine-2,6-dicarboxylic acid), was obtained by reaction of Ca(NO_3_)_2_·4H_2_O with the proton-transfer compound (tataH)_2_(pydc) in aqueous solution. The [Ca(pydc)_3_]^4−^ anion has twofold crystallographic symmetry. It is a nine-coordinate Ca^II^ complex with a distorted tricapped trigonal-prismatic coordination geometry. The structure also contains four tataH^+^ cations and six uncoordinated water mol­ecules. There are extensive O—H⋯O, O—H⋯N, N—H⋯O, N—H⋯N and C—H⋯O hydrogen bonds in the crystal structure.

## Related literature

For related literature, see: Aghabozorg *et al.* (2006 ▶); Aghabozorg, Attar Gharamaleki *et al.* (2008 ▶); Aghabozorg, Manteghi & Sheshmani (2008 ▶); Aghajani *et al.* (2006 ▶); Sharif *et al.* (2007 ▶).
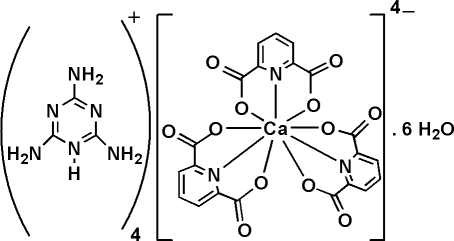


## Experimental

### 

#### Crystal data

(C_3_H_7_N_6_)_4_[Ca(C_7_H_3_NO_4_)_3_]·6H_2_O
*M* *_r_* = 1152.07
Monoclinic, 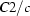

*a* = 17.9605 (15) Å
*b* = 10.1672 (9) Å
*c* = 25.922 (2) Å
β = 94.467 (2)°
*V* = 4719.1 (7) Å^3^
*Z* = 4
Mo *K*α radiation
μ = 0.24 mm^−1^
*T* = 100 (2) K
0.30 × 0.20 × 0.20 mm

#### Data collection

Bruker SMART APEXII diffractometer
Absorption correction: multi-scan (*SADABS*; Sheldrick, 1996 ▶) *T*_min_ = 0.928, *T*_max_ = 0.954
16983 measured reflections
6215 independent reflections
4412 reflections with *I* > 2σ(*I*)
*R*_int_ = 0.031

#### Refinement

*R*[*F* ^2^ > 2σ(*F* ^2^)] = 0.051
*wR*(*F* ^2^) = 0.127
*S* = 1.09
6215 reflections
365 parameters
H-atom parameters constrained
Δρ_max_ = 0.51 e Å^−3^
Δρ_min_ = −0.77 e Å^−3^

### 

Data collection: *APEX2* (Bruker, 2007 ▶); cell refinement: *SAINT* (Bruker, 2007 ▶); data reduction: *SAINT*; program(s) used to solve structure: *SHELXS97* (Sheldrick, 2008 ▶); program(s) used to refine structure: *SHELXL97* (Sheldrick, 2008 ▶); molecular graphics: *SHELXTL* (Sheldrick, 2008 ▶) and *Mercury* (Macrae *et al.*, 2006 ▶); software used to prepare material for publication: *SHELXL97*.

## Supplementary Material

Crystal structure: contains datablocks I, global. DOI: 10.1107/S1600536808022873/om2249sup1.cif

Structure factors: contains datablocks I. DOI: 10.1107/S1600536808022873/om2249Isup2.hkl

Additional supplementary materials:  crystallographic information; 3D view; checkCIF report

## Figures and Tables

**Table 1:** Hydrogen-bond geometry (Å, °)

*D*—H⋯*A*	*D*—H	H⋯*A*	*D*⋯*A*	*D*—H⋯*A*
O1*W*—H1*WB*⋯O2^i^	0.84	1.91	2.710 (3)	160
O1*W*—H1*WA*⋯O6^ii^	0.84	1.92	2.751 (3)	170
O2*W*—H2*WB*⋯N4^iii^	0.84	2.26	3.022 (3)	151
N3—H3*N*⋯O2	0.88	1.79	2.667 (2)	176
O2*W*—H2*WA*⋯O5	0.84	2.05	2.888 (3)	175
O3*W*—H3*WB*⋯O2*W*^iv^	0.84	2.18	2.963 (3)	154
O3*W*—H3*WA*⋯N14^v^	0.84	2.17	3.007 (3)	180
N6—H6*NA*⋯O1*W*^i^	0.88	2.45	3.294 (3)	162
N6—H6*NB*⋯O1*W*	0.88	1.90	2.740 (3)	160
N7—H7*NA*⋯N11^v^	0.88	2.22	3.103 (3)	176
N7—H7*NB*⋯O2*W*^ii^	0.88	2.44	3.205 (3)	146
N7—H7*NB*⋯O4^ii^	0.88	2.53	3.111 (2)	124
N8—H8*NA*⋯O1	0.88	2.07	2.948 (2)	172
N12—H11*A*⋯N10^vi^	0.88	2.23	3.109 (2)	178
N12—H11*B*⋯O5	0.88	2.32	3.196 (2)	175
N8—H8*NB*⋯O3*W*	0.88	2.15	2.832 (4)	134
N9—H9*NA*⋯O3	0.88	1.89	2.754 (2)	165
N13—H13*A*⋯O4^vii^	0.88	2.08	2.925 (2)	160
N13—H13*B*⋯O6^vi^	0.88	1.96	2.790 (2)	157
N14—H14*A*⋯O3	0.88	2.39	3.143 (2)	144
N14—H14*B*⋯N5^viii^	0.88	2.10	2.978 (3)	178
C4—H4*A*⋯O2^ix^	0.95	2.55	3.370 (3)	145

Symmetry codes: (i) 
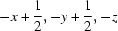
; (ii) 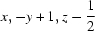; (iii) 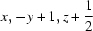; (iv) 
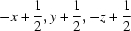
; (v) 
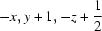
; (vi) 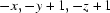; (vii) 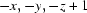; (viii) 
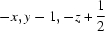
; (ix) 
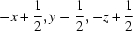
.

**Fig. 1.**
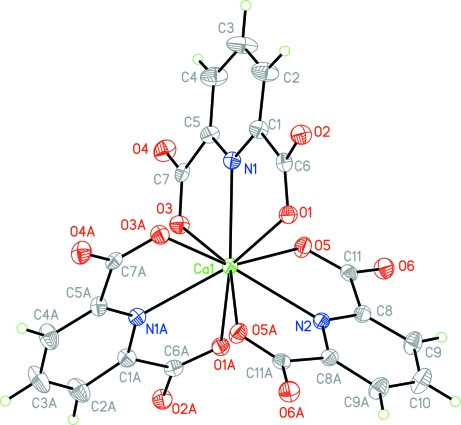
The molecular structure of the title compound, with displacement ellipsoids drawn at the 50% probability level. The anion is located on a 2-fold axis and thus, the asymmetric unit contains one half anion, two cations and three water molecules.

**Fig. 2.**
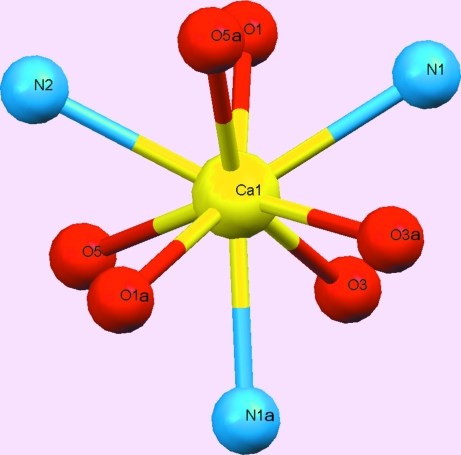
A view of the distorted tricapped trigonal prism around the CaII atom.

**Fig. 3.**
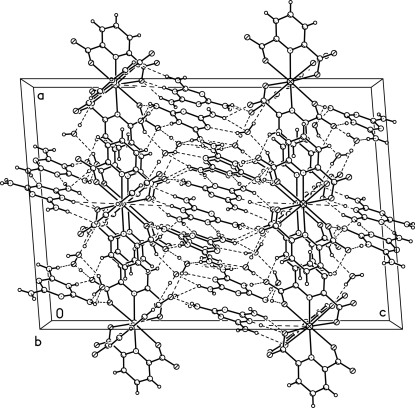
Crystal packing with hydrogen bonds shown as dashed lines.

## supplementary crystallographic information

### Comment

Hydrogen bonding plays a key role in chemical, catalytic and biochemical processes, as well as in supramolecular chemistry and crystal engineering. Recently we have reported the reaction between the proton-transfer compound, (tataH)_2_(pydc) with metal salts Co(NO_3_)_2_.6H_2_O, Bi(NO_3_)_3_.5H_2_O, Zn(NO_3_)_2_.4H_2_O and Cd(NO_3_)_2_.2H_2_O in a 2:1 molar ratio. These reactions lead to the formation of the (tataH)_2_[Co(H_2_O)_6_][Co(pydc)_2_]_2_.4H_2_O (Aghabozorg, Attar Gharamaleki *et al.*, 2008), (tataH)_n_ [Bi(pydc)_2_(H_2_O)]_n_(Sharif *et al.*, 2007), (tataH)_2_ [Zn(pydc)_2_].10H_2_O (Aghajani *et al.*, 2006) and (tataH)_2_ [Cd(pydc)_2_] (Aghabozorg, Aghajani *et al.*, 2006) compounds respectively. For more details and related literature see our recent review article (Aghabozorg, Manteghi *et al.*, 2008).

The structure of the title compound is shown in Fig.1. The anion has crystallographic 2-fold symmetry. The compound contains [Ca(pydc)_3_]^4–^ anion, four (tataH)^+^ cations and six uncoordinated water molecules. In the [Ca(pydc)_3_]^4–^ anions, Ca^II^ atom is nine-coordinated by three N atoms (N1, N1a and N2) and six O atoms (O1, O1a, O3, O3a, O5 and O5a) with the range of 2.5031 (14)–2.5472 (15)Å from the carboxylate groups of three (pydc)^2–^ groups that act as tridentate ligands. The coordination geometry around the Ca^II^ atom is distorted tricapped trigonal prism (Fig.2). Three N atoms (N1, N1a and N2) occupying three cap positions and make a flat triangle with N1—Ca1—N1a: 119.78 (8)°, N1—Ca1—N2: 120.11 (4)° and N1a—Ca1—N2: 120.11 (4)° that the sum of these angles is 360.00° and six O atoms (O1, O1a, O3, O3a, O5 and O5a) forming the trigonal prism positions.

There are various hydrogen bonds such as O—H···O, O—H···N, N—H···O and N—H···N [in the range 2.667 (2)–3.294 (3) Å] in this structure (Fig.3) and C—H···O hydrogen bonds [with D···A 3.370 (3) Å] are also present (Table 1). These extensive hydrogen bonds between [Ca(pydc)_3_]^4–^ anions, (tataH)^+^ cations and uncoordinated water molecules play an important role in stabilization of the crystal packing.

### Experimental

The proton-transfer compound, (tataH)_2_(pydc), was prepared by the reaction of pyridine-2,6-dicarboxylic acid (pydcH_2_) with 2,4,6-triamino-1,3,5-triazine (tata). The reaction between Ca(NO_3_)_2_.4H_2_O (118 mg, 0.5 mmol) in water (20 ml) and (tataH)_2_(pydc) (420 mg, 1.0 mmol) in water (20 ml), in 1:2 molar ratio gave a colorless compound after slow evaporation of the solvent at the room temperature.

### Refinement

The hydrogen atoms of NH groups and also H atoms of water molecule were found in difference Fourier synthesis. The hydrogen atoms of the H(C) atom positions were calculated. All hydrogen atoms were refined in isotropic approximation in riding model with the *U*_iso_(H) parameters equal to 1.2 *U*_eq_(Xi), where U(Xi) the equivalent thermal parameters of the carbon or nitrogen or oxygen atom to which corresponding H atom is bonded. Distances employed in the riding model are, N-H, 0.88 Å; O-H, 0.84 Å; C-H, 0.95 Å.

### Crystal data

**Table:**

(C_3_H_7_N_6_)_4_[Ca(C_7_H_3_N_1_O_4_)_3_]·6H_2_O	*F*_000_ = 2400
*M**_r_* = 1152.07	*D*_x_ = 1.622 Mg m^−^^3^
Monoclinic, *C*2/*c*	Mo *K*α radiation λ = 0.71073 Å
Hall symbol: -C 2yc	Cell parameters from 935 reflections
*a* = 17.9605 (15) Å	θ = 3–29º
*b* = 10.1672 (9) Å	µ = 0.24 mm^−^^1^
*c* = 25.922 (2) Å	*T* = 100 (2) K
β = 94.467 (2)º	Prism, colourless
*V* = 4719.1 (7) Å^3^	0.30 × 0.20 × 0.20 mm
*Z* = 4	

### Data collection

**Table:**

Bruker SMART APEXII diffractometer	6215 independent reflections
Radiation source: fine-focus sealed tube	4412 reflections with *I* > 2σ(*I*)
Monochromator: graphite	*R*_int_ = 0.031
*T* = 100(2) K	θ_max_ = 29.0º
ω scans	θ_min_ = 1.6º
Absorption correction: multi-scan(SADABS; Sheldrick, 1996)	*h* = −19→24
*T*_min_ = 0.928, *T*_max_ = 0.954	*k* = −13→13
16983 measured reflections	*l* = −35→35

### Refinement

**Table:**

Refinement on *F*^2^	Secondary atom site location: difference Fourier map
Least-squares matrix: full	Hydrogen site location: inferred from neighbouring sites
*R*[*F*^2^ > 2σ(*F*^2^)] = 0.051	H-atom parameters constrained
*wR*(*F*^2^) = 0.127	*w* = 1/[σ^2^(*F*_o_^2^) + (0.0392*P*)^2^ + 1*P*] where *P* = (*F*_o_^2^ + 2*F*_c_^2^)/3
*S* = 1.09	(Δ/σ)_max_ < 0.001
6215 reflections	Δρ_max_ = 0.51 e Å^−^^3^
365 parameters	Δρ_min_ = −0.77 e Å^−^^3^
Primary atom site location: structure-invariant direct methods	Extinction correction: none

### Special details

**Table:**

Geometry. All e.s.d.'s (except the e.s.d. in the dihedral angle between two l.s. planes) are estimated using the full covariance matrix. The cell e.s.d.'s are taken into account individually in the estimation of e.s.d.'s in distances, angles and torsion angles; correlations between e.s.d.'s in cell parameters are only used when they are defined by crystal symmetry. An approximate (isotropic) treatment of cell e.s.d.'s is used for estimating e.s.d.'s involving l.s. planes.
Refinement. Refinement of *F*^2^ against ALL reflections. The weighted *R*-factor *wR* and goodness of fit *S* are based on *F*^2^, conventional *R*-factors *R* are based on *F*, with *F* set to zero for negative *F*^2^. The threshold expression of *F*^2^ > σ(*F*^2^) is used only for calculating *R*-factors(gt) *etc*. and is not relevant to the choice of reflections for refinement. *R*-factors based on *F*^2^ are statistically about twice as large as those based on *F*, and *R*- factors based on ALL data will be even larger.

### Fractional atomic coordinates and isotropic or equivalent isotropic displacement parameters (Å^2^)

**Table:**

	*x*	*y*	*z*	*U*_iso_*/*U*_eq_	
Ca1	0.0000	0.32225 (5)	0.2500	0.01468 (13)	
C1	0.17008 (12)	0.2206 (2)	0.21823 (8)	0.0189 (4)	
N1	0.12161 (10)	0.19798 (16)	0.25401 (6)	0.0167 (3)	
O1	0.08882 (8)	0.38891 (14)	0.18496 (5)	0.0198 (3)	
O1W	0.25538 (11)	0.3625 (2)	−0.08194 (7)	0.0511 (6)	
H1WB	0.2800	0.3196	−0.1025	0.061*	
H1WA	0.2150	0.3792	−0.0992	0.061*	
O2W	0.20543 (11)	0.3177 (2)	0.38198 (7)	0.0464 (5)	
H2WB	0.2093	0.3575	0.4105	0.056*	
H2WA	0.1704	0.3502	0.3627	0.056*	
O2	0.18167 (9)	0.32683 (15)	0.13789 (6)	0.0225 (3)	
N2	0.0000	0.5723 (2)	0.2500	0.0160 (5)	
C2	0.23993 (13)	0.1634 (3)	0.21999 (9)	0.0291 (5)	
H2A	0.2733	0.1824	0.1943	0.035*	
O3W	0.16530 (16)	0.8783 (3)	0.17830 (10)	0.0743 (8)	
H3WB	0.2094	0.8677	0.1704	0.089*	
H3WA	0.1367	0.9132	0.1550	0.089*	
N3	0.16153 (10)	0.53273 (18)	0.07458 (7)	0.0222 (4)	
H3N	0.1666	0.4663	0.0963	0.027*	
O3	0.02261 (8)	0.15716 (14)	0.32346 (5)	0.0203 (3)	
C3	0.26030 (14)	0.0777 (3)	0.26017 (9)	0.0345 (6)	
H3A	0.3079	0.0366	0.2624	0.041*	
O4	0.10324 (9)	0.02965 (15)	0.37134 (6)	0.0252 (3)	
C4	0.21035 (14)	0.0525 (2)	0.29714 (9)	0.0292 (5)	
H4A	0.2230	−0.0063	0.3249	0.035*	
N4	0.18475 (10)	0.62366 (18)	−0.00573 (7)	0.0217 (4)	
O5	0.08073 (9)	0.43271 (14)	0.32086 (6)	0.0220 (3)	
C5	0.14127 (12)	0.1153 (2)	0.29278 (8)	0.0183 (4)	
N5	0.12433 (10)	0.75024 (17)	0.05810 (7)	0.0207 (4)	
N6	0.22996 (11)	0.41835 (19)	0.01860 (8)	0.0275 (4)	
H6NA	0.2338	0.3554	0.0419	0.033*	
H6NB	0.2477	0.4118	−0.0120	0.033*	
O6	0.11801 (9)	0.61386 (15)	0.36402 (6)	0.0236 (3)	
C6	0.14421 (12)	0.3194 (2)	0.17714 (7)	0.0174 (4)	
N7	0.13887 (10)	0.83102 (18)	−0.02277 (7)	0.0224 (4)	
H7NA	0.1188	0.9049	−0.0129	0.027*	
H7NB	0.1526	0.8246	−0.0545	0.027*	
C7	0.08494 (12)	0.09839 (19)	0.33279 (8)	0.0171 (4)	
N8	0.09931 (11)	0.64715 (19)	0.13431 (7)	0.0257 (4)	
H8NA	0.0972	0.5743	0.1525	0.031*	
H8NB	0.0923	0.7219	0.1503	0.031*	
C8	0.04098 (11)	0.6393 (2)	0.28661 (8)	0.0175 (4)	
N9	−0.02526 (10)	0.21627 (17)	0.41916 (6)	0.0189 (4)	
H9NA	−0.0085	0.2124	0.3882	0.023*	
C9	0.04259 (14)	0.7762 (2)	0.28768 (9)	0.0250 (5)	
H9A	0.0724	0.8215	0.3138	0.030*	
C10	0.0000	0.8452 (3)	0.2500	0.0301 (8)	
H10A	0.0000	0.9386	0.2500	0.036*	
N10	−0.03939 (10)	0.32331 (17)	0.49808 (6)	0.0179 (4)	
C11	0.08390 (11)	0.5555 (2)	0.32701 (8)	0.0170 (4)	
N11	−0.07504 (10)	0.09514 (17)	0.48552 (7)	0.0187 (4)	
N12	0.01468 (10)	0.42995 (18)	0.43181 (7)	0.0219 (4)	
H11A	0.0226	0.4986	0.4522	0.041 (8)*	
H11B	0.0304	0.4273	0.4005	0.033 (7)*	
C12	0.15006 (12)	0.7326 (2)	0.01068 (8)	0.0199 (4)	
N13	−0.08510 (11)	0.19908 (18)	0.56239 (7)	0.0230 (4)	
H13A	−0.0983	0.1240	0.5759	0.039 (8)*	
H13B	−0.0842	0.2683	0.5828	0.029 (7)*	
C13	0.12875 (12)	0.6464 (2)	0.08866 (8)	0.0206 (4)	
N14	−0.06375 (11)	0.00349 (19)	0.40544 (7)	0.0273 (4)	
H14A	−0.0459	0.0126	0.3750	0.033 (7)*	
H14B	−0.0830	−0.0706	0.4158	0.036 (8)*	
C14	0.19208 (12)	0.5258 (2)	0.02825 (8)	0.0217 (4)	
C15	−0.01687 (11)	0.3235 (2)	0.45053 (8)	0.0171 (4)	
C16	−0.06599 (12)	0.2060 (2)	0.51433 (8)	0.0183 (4)	
C17	−0.05485 (11)	0.1040 (2)	0.43758 (8)	0.0188 (4)	

### Atomic displacement parameters (Å^2^)

**Table:**

	*U*^11^	*U*^22^	*U*^33^	*U*^12^	*U*^13^	*U*^23^
Ca1	0.0160 (3)	0.0136 (3)	0.0145 (3)	0.000	0.0015 (2)	0.000
C1	0.0193 (11)	0.0216 (10)	0.0160 (9)	0.0004 (8)	0.0023 (8)	0.0019 (8)
N1	0.0178 (9)	0.0173 (8)	0.0150 (8)	−0.0001 (7)	0.0020 (6)	−0.0004 (6)
O1	0.0198 (8)	0.0186 (7)	0.0215 (7)	0.0008 (6)	0.0044 (6)	0.0036 (6)
O1W	0.0303 (10)	0.0852 (16)	0.0357 (10)	0.0236 (10)	−0.0111 (8)	−0.0261 (10)
O2W	0.0510 (12)	0.0472 (12)	0.0378 (10)	0.0222 (10)	−0.0160 (9)	−0.0154 (9)
O2	0.0240 (8)	0.0243 (8)	0.0200 (7)	0.0016 (6)	0.0077 (6)	0.0054 (6)
N2	0.0160 (12)	0.0156 (11)	0.0168 (11)	0.000	0.0044 (9)	0.000
C2	0.0240 (12)	0.0416 (14)	0.0227 (11)	0.0080 (11)	0.0074 (9)	0.0077 (10)
O3W	0.089 (2)	0.0615 (16)	0.0691 (17)	0.0005 (15)	−0.0175 (14)	−0.0047 (13)
N3	0.0229 (10)	0.0190 (9)	0.0250 (9)	0.0023 (7)	0.0041 (8)	0.0065 (7)
O3	0.0202 (8)	0.0223 (8)	0.0187 (7)	0.0012 (6)	0.0037 (6)	0.0027 (6)
C3	0.0242 (13)	0.0500 (16)	0.0300 (13)	0.0167 (11)	0.0075 (10)	0.0120 (11)
O4	0.0322 (9)	0.0242 (8)	0.0197 (7)	0.0030 (7)	0.0047 (6)	0.0077 (6)
C4	0.0303 (13)	0.0347 (13)	0.0227 (11)	0.0120 (11)	0.0038 (9)	0.0097 (10)
N4	0.0190 (9)	0.0227 (9)	0.0230 (9)	0.0009 (7)	0.0003 (7)	0.0035 (7)
O5	0.0249 (8)	0.0161 (7)	0.0238 (8)	−0.0007 (6)	−0.0048 (6)	−0.0004 (6)
C5	0.0209 (11)	0.0182 (10)	0.0160 (9)	0.0029 (8)	0.0022 (8)	0.0008 (7)
N5	0.0205 (9)	0.0192 (9)	0.0220 (9)	−0.0017 (7)	0.0004 (7)	0.0015 (7)
N6	0.0297 (11)	0.0248 (10)	0.0290 (10)	0.0063 (8)	0.0080 (8)	0.0063 (8)
O6	0.0250 (8)	0.0246 (8)	0.0205 (7)	−0.0011 (7)	−0.0031 (6)	−0.0052 (6)
C6	0.0188 (10)	0.0183 (10)	0.0149 (9)	−0.0030 (8)	0.0006 (8)	0.0002 (7)
N7	0.0233 (10)	0.0222 (9)	0.0217 (9)	0.0012 (8)	0.0006 (7)	0.0037 (7)
C7	0.0231 (11)	0.0117 (9)	0.0167 (9)	−0.0021 (8)	0.0029 (8)	−0.0013 (7)
N8	0.0339 (11)	0.0198 (9)	0.0241 (9)	0.0005 (8)	0.0065 (8)	0.0028 (7)
C8	0.0183 (10)	0.0171 (9)	0.0178 (9)	−0.0006 (8)	0.0055 (8)	−0.0011 (7)
N9	0.0219 (9)	0.0207 (9)	0.0147 (8)	0.0016 (7)	0.0050 (7)	0.0009 (7)
C9	0.0322 (13)	0.0194 (10)	0.0228 (11)	−0.0025 (9)	−0.0019 (9)	−0.0030 (8)
C10	0.044 (2)	0.0179 (15)	0.0273 (17)	0.000	−0.0032 (15)	0.000
N10	0.0215 (9)	0.0158 (8)	0.0167 (8)	0.0008 (7)	0.0032 (7)	0.0012 (6)
C11	0.0146 (10)	0.0199 (10)	0.0168 (9)	−0.0012 (8)	0.0034 (7)	−0.0010 (7)
N11	0.0197 (9)	0.0173 (8)	0.0193 (8)	−0.0011 (7)	0.0038 (7)	−0.0011 (7)
N12	0.0249 (10)	0.0214 (9)	0.0200 (9)	−0.0006 (8)	0.0068 (7)	0.0025 (7)
C12	0.0144 (10)	0.0228 (10)	0.0217 (10)	−0.0031 (8)	−0.0035 (8)	0.0027 (8)
N13	0.0356 (11)	0.0160 (9)	0.0182 (8)	−0.0043 (8)	0.0071 (8)	−0.0012 (7)
C13	0.0185 (10)	0.0201 (10)	0.0227 (10)	−0.0027 (8)	−0.0004 (8)	0.0012 (8)
N14	0.0353 (11)	0.0258 (10)	0.0221 (9)	−0.0079 (9)	0.0106 (8)	−0.0068 (8)
C14	0.0152 (10)	0.0234 (11)	0.0265 (11)	−0.0008 (8)	0.0009 (8)	0.0033 (8)
C15	0.0150 (10)	0.0172 (9)	0.0189 (9)	0.0024 (8)	0.0004 (7)	0.0021 (8)
C16	0.0170 (10)	0.0183 (10)	0.0197 (10)	0.0016 (8)	0.0014 (8)	0.0009 (8)
C17	0.0155 (10)	0.0206 (10)	0.0204 (10)	0.0015 (8)	0.0020 (8)	−0.0013 (8)

### Geometric parameters (Å, °)

**Table:**

Ca1—O1	2.5031 (14)	O5—C11	1.259 (2)
Ca1—O1^i^	2.5032 (14)	C5—C7	1.514 (3)
Ca1—O5^i^	2.5145 (15)	N5—C13	1.319 (3)
Ca1—O5	2.5145 (15)	N5—C12	1.358 (3)
Ca1—N1^i^	2.5184 (18)	N6—C14	1.321 (3)
Ca1—N1	2.5184 (18)	N6—H6NA	0.8800
Ca1—N2	2.542 (2)	N6—H6NB	0.8798
Ca1—O3^i^	2.5472 (15)	O6—C11	1.248 (2)
Ca1—O3	2.5472 (15)	N7—C12	1.329 (3)
C1—N1	1.340 (3)	N7—H7NA	0.8800
C1—C2	1.380 (3)	N7—H7NB	0.8800
C1—C6	1.511 (3)	N8—C13	1.333 (3)
N1—C5	1.337 (3)	N8—H8NA	0.8800
O1—C6	1.250 (2)	N8—H8NB	0.8800
O1W—H1WB	0.8400	C8—C9	1.393 (3)
O1W—H1WA	0.8400	C8—C11	1.514 (3)
O2W—H2WB	0.8400	N9—C15	1.362 (3)
O2W—H2WA	0.8400	N9—C17	1.361 (3)
O2—C6	1.265 (2)	N9—H9NA	0.8800
N2—C8^i^	1.340 (2)	C9—C10	1.384 (3)
N2—C8	1.340 (2)	C9—H9A	0.9500
C2—C3	1.385 (3)	C10—C9^i^	1.384 (3)
C2—H2A	0.9500	C10—H10A	0.9500
O3W—H3WB	0.8400	N10—C15	1.327 (3)
O3W—H3WA	0.8401	N10—C16	1.363 (3)
N3—C13	1.360 (3)	N11—C17	1.324 (3)
N3—C14	1.360 (3)	N11—C16	1.355 (3)
N3—H3N	0.8800	N12—C15	1.330 (3)
O3—C7	1.275 (3)	N12—H11A	0.8801
C3—C4	1.386 (3)	N12—H11B	0.8799
C3—H3A	0.9500	N13—C16	1.319 (3)
O4—C7	1.243 (2)	N13—H13A	0.8800
C4—C5	1.392 (3)	N13—H13B	0.8799
C4—H4A	0.9500	N14—C17	1.320 (3)
N4—C14	1.329 (3)	N14—H14A	0.8800
N4—C12	1.356 (3)	N14—H14B	0.8800
			
O1—Ca1—O1^i^	148.58 (7)	N1—C5—C4	122.08 (19)
O1—Ca1—O5^i^	75.43 (5)	N1—C5—C7	115.77 (18)
O1^i^—Ca1—O5^i^	90.55 (5)	C4—C5—C7	122.11 (19)
O1—Ca1—O5	90.55 (5)	C13—N5—C12	115.43 (19)
O1^i^—Ca1—O5	75.43 (5)	C14—N6—H6NA	119.0
O5^i^—Ca1—O5	126.94 (7)	C14—N6—H6NB	117.5
O1—Ca1—N1^i^	134.76 (5)	H6NA—N6—H6NB	123.4
O1^i^—Ca1—N1^i^	64.37 (5)	O1—C6—O2	125.19 (19)
O5^i^—Ca1—N1^i^	75.23 (5)	O1—C6—C1	117.84 (17)
O5—Ca1—N1^i^	134.68 (5)	O2—C6—C1	116.96 (18)
O1—Ca1—N1	64.37 (5)	C12—N7—H7NA	119.8
O1^i^—Ca1—N1	134.76 (5)	C12—N7—H7NB	121.1
O5^i^—Ca1—N1	134.68 (5)	H7NA—N7—H7NB	119.1
O5—Ca1—N1	75.23 (5)	O4—C7—O3	126.11 (19)
N1^i^—Ca1—N1	119.78 (8)	O4—C7—C5	117.75 (19)
O1—Ca1—N2	74.29 (4)	O3—C7—C5	116.14 (17)
O1^i^—Ca1—N2	74.29 (4)	C13—N8—H8NA	120.4
O5^i^—Ca1—N2	63.47 (4)	C13—N8—H8NB	120.4
O5—Ca1—N2	63.47 (4)	H8NA—N8—H8NB	117.4
N1^i^—Ca1—N2	120.11 (4)	N2—C8—C9	122.1 (2)
N1—Ca1—N2	120.11 (4)	N2—C8—C11	115.19 (18)
O1—Ca1—O3^i^	75.39 (5)	C9—C8—C11	122.70 (19)
O1^i^—Ca1—O3^i^	127.53 (5)	C15—N9—C17	119.35 (17)
O5^i^—Ca1—O3^i^	72.48 (5)	C15—N9—H9NA	123.4
O5—Ca1—O3^i^	152.84 (5)	C17—N9—H9NA	117.0
N1^i^—Ca1—O3^i^	63.34 (5)	C10—C9—C8	118.9 (2)
N1—Ca1—O3^i^	77.73 (5)	C10—C9—H9A	120.6
N2—Ca1—O3^i^	131.22 (3)	C8—C9—H9A	120.6
O1—Ca1—O3	127.53 (5)	C9^i^—C10—C9	119.1 (3)
O1^i^—Ca1—O3	75.39 (5)	C9^i^—C10—H10A	120.5
O5^i^—Ca1—O3	152.84 (5)	C9—C10—H10A	120.5
O5—Ca1—O3	72.48 (5)	C15—N10—C16	115.35 (18)
N1^i^—Ca1—O3	77.73 (5)	O6—C11—O5	125.73 (19)
N1—Ca1—O3	63.34 (5)	O6—C11—C8	117.19 (18)
N2—Ca1—O3	131.22 (3)	O5—C11—C8	117.06 (18)
O3^i^—Ca1—O3	97.56 (7)	C17—N11—C16	115.51 (18)
N1—C1—C2	122.86 (19)	C15—N12—H11A	118.7
N1—C1—C6	114.91 (18)	C15—N12—H11B	119.2
C2—C1—C6	122.16 (19)	H11A—N12—H11B	121.9
C5—N1—C1	118.70 (18)	N7—C12—N4	117.41 (19)
C5—N1—Ca1	121.61 (13)	N7—C12—N5	116.5 (2)
C1—N1—Ca1	119.56 (13)	N4—C12—N5	126.05 (19)
C6—O1—Ca1	120.86 (12)	C16—N13—H13A	121.4
H1WB—O1W—H1WA	104.1	C16—N13—H13B	122.1
H2WB—O2W—H2WA	109.9	H13A—N13—H13B	116.5
C8^i^—N2—C8	118.9 (2)	N5—C13—N8	121.3 (2)
C8^i^—N2—Ca1	120.53 (12)	N5—C13—N3	121.8 (2)
C8—N2—Ca1	120.53 (12)	N8—C13—N3	116.96 (19)
C3—C2—C1	118.4 (2)	C17—N14—H14A	116.7
C3—C2—H2A	120.8	C17—N14—H14B	120.1
C1—C2—H2A	120.8	H14A—N14—H14B	123.0
H3WB—O3W—H3WA	114.4	N6—C14—N4	121.3 (2)
C13—N3—C14	119.60 (18)	N6—C14—N3	117.3 (2)
C13—N3—H3N	120.4	N4—C14—N3	121.4 (2)
C14—N3—H3N	119.8	N10—C15—N12	120.64 (19)
C7—O3—Ca1	122.48 (12)	N10—C15—N9	121.73 (18)
C2—C3—C4	119.2 (2)	N12—C15—N9	117.63 (18)
C2—C3—H3A	120.4	N13—C16—N11	116.54 (19)
C4—C3—H3A	120.4	N13—C16—N10	117.51 (19)
C3—C4—C5	118.7 (2)	N11—C16—N10	125.96 (18)
C3—C4—H4A	120.7	N14—C17—N11	120.7 (2)
C5—C4—H4A	120.7	N14—C17—N9	117.33 (19)
C14—N4—C12	115.36 (18)	N11—C17—N9	121.95 (19)
C11—O5—Ca1	123.58 (13)		
			
C2—C1—N1—C5	−0.8 (3)	N1—Ca1—O5—C11	138.44 (17)
C6—C1—N1—C5	−177.72 (18)	N2—Ca1—O5—C11	3.08 (14)
C2—C1—N1—Ca1	175.25 (18)	O3^i^—Ca1—O5—C11	132.90 (16)
C6—C1—N1—Ca1	−1.6 (2)	O3—Ca1—O5—C11	−155.35 (17)
O1—Ca1—N1—C5	171.11 (17)	C1—N1—C5—C4	0.2 (3)
O1^i^—Ca1—N1—C5	22.09 (19)	Ca1—N1—C5—C4	−175.81 (17)
O5^i^—Ca1—N1—C5	−158.97 (14)	C1—N1—C5—C7	177.82 (18)
O5—Ca1—N1—C5	73.20 (15)	Ca1—N1—C5—C7	1.8 (2)
N1^i^—Ca1—N1—C5	−60.19 (14)	C3—C4—C5—N1	0.4 (4)
N2—Ca1—N1—C5	119.81 (14)	C3—C4—C5—C7	−177.1 (2)
O3^i^—Ca1—N1—C5	−109.38 (16)	Ca1—O1—C6—O2	162.52 (16)
O3—Ca1—N1—C5	−4.32 (14)	Ca1—O1—C6—C1	−19.1 (2)
O1—Ca1—N1—C1	−4.88 (14)	N1—C1—C6—O1	13.5 (3)
O1^i^—Ca1—N1—C1	−153.89 (14)	C2—C1—C6—O1	−163.4 (2)
O5^i^—Ca1—N1—C1	25.05 (18)	N1—C1—C6—O2	−167.97 (18)
O5—Ca1—N1—C1	−102.78 (15)	C2—C1—C6—O2	15.1 (3)
N1^i^—Ca1—N1—C1	123.83 (16)	Ca1—O3—C7—O4	170.52 (16)
N2—Ca1—N1—C1	−56.17 (16)	Ca1—O3—C7—C5	−9.4 (2)
O3^i^—Ca1—N1—C1	74.64 (15)	N1—C5—C7—O4	−175.02 (18)
O3—Ca1—N1—C1	179.70 (17)	C4—C5—C7—O4	2.6 (3)
O1^i^—Ca1—O1—C6	148.51 (15)	N1—C5—C7—O3	4.9 (3)
O5^i^—Ca1—O1—C6	−145.46 (15)	C4—C5—C7—O3	−177.5 (2)
O5—Ca1—O1—C6	86.33 (15)	C8^i^—N2—C8—C9	−0.29 (15)
N1^i^—Ca1—O1—C6	−94.42 (16)	Ca1—N2—C8—C9	179.71 (15)
N1—Ca1—O1—C6	13.04 (14)	C8^i^—N2—C8—C11	178.57 (19)
N2—Ca1—O1—C6	148.51 (15)	Ca1—N2—C8—C11	−1.43 (19)
O3^i^—Ca1—O1—C6	−70.16 (15)	N2—C8—C9—C10	0.6 (3)
O3—Ca1—O1—C6	18.19 (17)	C11—C8—C9—C10	−178.21 (17)
O1—Ca1—N2—C8^i^	80.76 (10)	C8—C9—C10—C9^i^	−0.27 (14)
O1^i^—Ca1—N2—C8^i^	−99.24 (10)	Ca1—O5—C11—O6	173.48 (15)
O5^i^—Ca1—N2—C8^i^	−0.53 (11)	Ca1—O5—C11—C8	−5.0 (2)
O5—Ca1—N2—C8^i^	179.47 (11)	N2—C8—C11—O6	−174.49 (16)
N1^i^—Ca1—N2—C8^i^	−52.29 (11)	C9—C8—C11—O6	4.4 (3)
N1—Ca1—N2—C8^i^	127.71 (11)	N2—C8—C11—O5	4.1 (3)
O3^i^—Ca1—N2—C8^i^	27.26 (11)	C9—C8—C11—O5	−177.1 (2)
O3—Ca1—N2—C8^i^	−152.74 (11)	C14—N4—C12—N7	−177.40 (19)
O1—Ca1—N2—C8	−99.24 (10)	C14—N4—C12—N5	1.4 (3)
O1^i^—Ca1—N2—C8	80.76 (10)	C13—N5—C12—N7	172.95 (19)
O5^i^—Ca1—N2—C8	179.47 (11)	C13—N5—C12—N4	−5.9 (3)
O5—Ca1—N2—C8	−0.53 (11)	C12—N5—C13—N8	−173.9 (2)
N1^i^—Ca1—N2—C8	127.71 (11)	C12—N5—C13—N3	4.3 (3)
N1—Ca1—N2—C8	−52.29 (11)	C14—N3—C13—N5	1.3 (3)
O3^i^—Ca1—N2—C8	−152.74 (11)	C14—N3—C13—N8	179.6 (2)
O3—Ca1—N2—C8	27.26 (11)	C12—N4—C14—N6	−174.6 (2)
N1—C1—C2—C3	0.8 (4)	C12—N4—C14—N3	4.7 (3)
C6—C1—C2—C3	177.5 (2)	C13—N3—C14—N6	173.2 (2)
O1—Ca1—O3—C7	2.23 (17)	C13—N3—C14—N4	−6.2 (3)
O1^i^—Ca1—O3—C7	−153.52 (15)	C16—N10—C15—N12	−175.54 (19)
O5^i^—Ca1—O3—C7	145.59 (14)	C16—N10—C15—N9	4.7 (3)
O5—Ca1—O3—C7	−74.48 (15)	C17—N9—C15—N10	−2.6 (3)
N1^i^—Ca1—O3—C7	140.11 (15)	C17—N9—C15—N12	177.67 (18)
N1—Ca1—O3—C7	7.43 (14)	C17—N11—C16—N13	−179.39 (19)
N2—Ca1—O3—C7	−100.42 (14)	C17—N11—C16—N10	0.9 (3)
O3^i^—Ca1—O3—C7	79.58 (14)	C15—N10—C16—N13	176.27 (19)
C1—C2—C3—C4	−0.2 (4)	C15—N10—C16—N11	−4.0 (3)
C2—C3—C4—C5	−0.4 (4)	C16—N11—C17—N14	−177.5 (2)
O1—Ca1—O5—C11	75.17 (16)	C16—N11—C17—N9	1.6 (3)
O1^i^—Ca1—O5—C11	−76.38 (16)	C15—N9—C17—N14	178.31 (19)
O5^i^—Ca1—O5—C11	3.08 (14)	C15—N9—C17—N11	−0.8 (3)
N1^i^—Ca1—O5—C11	−104.08 (16)		

Symmetry codes: (i) −*x*, *y*, −*z*+1/2.

### Hydrogen-bond geometry (Å, °)

**Table:**

*D*—H···*A*	*D*—H	H···*A*	*D*···*A*	*D*—H···*A*
O1W—H1WB···O2^ii^	0.84	1.91	2.710 (3)	160
O1W—H1WA···O6^iii^	0.84	1.92	2.751 (3)	170
O2W—H2WB···N4^iv^	0.84	2.26	3.022 (3)	151
N3—H3N···O2	0.88	1.79	2.667 (2)	176
O2W—H2WA···O5	0.84	2.05	2.888 (3)	175
O3W—H3WB···O2W^v^	0.84	2.18	2.963 (3)	154
O3W—H3WA···N14^vi^	0.84	2.17	3.007 (3)	180
N6—H6NA···O1W^ii^	0.88	2.45	3.294 (3)	162
N6—H6NB···O1W	0.88	1.90	2.740 (3)	160
N7—H7NA···N11^vi^	0.88	2.22	3.103 (3)	176
N7—H7NB···O2W^iii^	0.88	2.44	3.205 (3)	146
N7—H7NB···O4^iii^	0.88	2.53	3.111 (2)	124
N8—H8NA···O1	0.88	2.07	2.948 (2)	172
N12—H11A···N10^vii^	0.88	2.23	3.109 (2)	178
N12—H11B···O5	0.88	2.32	3.196 (2)	175
N8—H8NB···O3W	0.88	2.15	2.832 (4)	134
N9—H9NA···O3	0.88	1.89	2.754 (2)	165
N13—H13A···O4^viii^	0.88	2.08	2.925 (2)	160
N13—H13B···O6^vii^	0.88	1.96	2.790 (2)	157
N14—H14A···O3	0.88	2.39	3.143 (2)	144
N14—H14B···N5^ix^	0.88	2.10	2.978 (3)	178
C4—H4A···O2^x^	0.95	2.55	3.370 (3)	145

Symmetry codes: (ii) −*x*+1/2, −*y*+1/2, −*z*; (iii) *x*, −*y*+1, *z*−1/2; (iv) *x*, −*y*+1, *z*+1/2;  (v) −*x*+1/2, *y*+1/2, −*z*+1/2; (vi) −*x*, *y*+1, −*z*+1/2; (vii) −*x*, −*y*+1, −*z*+1; (viii) −*x*, −*y*, −*z*+1; (ix) −*x*, *y*−1, −*z*+1/2; (x) −*x*+1/2, *y*−1/2, −*z*+1/2.
